# The effect of perceived value on farmers’ livestock manure resource utilization behavior: Evidence from Shandong, China

**DOI:** 10.3389/fpsyg.2023.1098587

**Published:** 2023-02-09

**Authors:** Runze Gao, Guoliang Liu, Yuze Fan, Xueyang Wang, Zhong Ren

**Affiliations:** Business School, Shandong Normal University, Jinan, China

**Keywords:** perceived value, livestock manure, resource utilization of manure, Concurrent business, Multi-group SEM

## Abstract

**Introduction:**

The rapid development of animal husbandry has brought many problems such as ecological environmental pollution and public health damage. The resource utilization of livestock manure is the key way to deal with the above crisis and turn waste into treasure.

**Methods:**

Based on the theory of perceived value, this paper uses multi-group structural equation model to explore the driving mechanism of perceived value on the resource utilization behavior of livestock manure.

**Results and discussion:**

The results showed that: (1) The resource utilization behavior of livestock manure followed the logic of “cognitive level → cognitive trade-off → perceived value → behavioral intention → behavioral performance.” Perceived benefit and perceived risk have positive and reverse driving effects on perceived value, respectively. Perceived value has a positive driving effect on behavioral intention. The behavioral intention has a positive driving effect on utilization behavior. (2) Among the observed variables of perceived benefits, ecological benefits have the greatest impact; Among the observed variables of perceived risk, economic risk has the greatest impact. Among the observed variables of perceived value, Significance cognition has the greatest influence. Among the observed variables of behavioral intention, utilization intention has the greatest influence. (3) The perceived value has a differential effect on the utilization behavior of livestock manure resources of different part-time farmers, and the driving effect is more obvious for full-time farmers.

**Conclusions:**

Therefore, it is necessary to improve the resource utilization system of livestock manure, increase the channel for realizing the output of manure resources, strengthen technical assistance and policy subsidies, and implement policies according to local conditions to improve the overall perceived value of farmers.

## 1. Introduction

The rapid development of global animal husbandry not only meets the needs of a high-quality diet but also produces a large amount of livestock manure. Livestock manure, as the focus of agricultural non-point source pollution prevention and control (Li et al., 2019), will not only produce air pollution, soil destruction, water quality deterioration, and other environmental problems but also cause harm to human health. Studies have found that in low- and middle-income countries, livestock manure pollution is the main cause of gastrointestinal diseases in domestic farmers for many years (Delahoy et al., 2018).

However, livestock manure is not all bad. Relevant studies have shown that organic matter, nitrogen, phosphorus, potassium, and other components rich in livestock manure can not only provide nutrients needed for crop growth but also generate a large amount of electricity and gas energy through biogas and other projects (Holm-Nielsen et al., 2009; Ramos-Suárez et al., 2019; Li J. et al., 2021). It can be seen that strengthening the resource utilization of livestock manure is of great significance to the high-quality development of the ecological environment and the sustainable development of agriculture. The Chinese government attaches great importance to the resource utilization of livestock manure and has issued a series of policy notices, such as “On Further Clarifying the Requirements for Returning Livestock manure to the Field and Strengthening Supervision of Livestock manure Pollution” and “Opinions on Promoting the Resource Utilization of Livestock manure,” to accelerate the resource utilization of livestock manure. According to statistics from the Ministry of Agriculture and Rural Affairs of China, the utilization rate of livestock manure resources in China reached 75% in 2020 (Website of the Ministry of Agriculture and Rural Affairs, 2020). However, as the world’s largest livestock producer, with an annual output of 3.8 billion tons of livestock manure, China still faces great ecological and environmental pressure. The Chinese government aims to use 80% of livestock manure by 2025. Therefore, there is still a long way to go to continuously promote the utilization rate of livestock manure resources.

As the main body of livestock manure resource utilization, the behavioral intention of farmers will directly affect the progress of livestock manure resource utilization. There have been many studies on the willingness or behavior of livestock manure resource utilization by farmers in academia, which can be roughly divided into three aspects: individual and family characteristics, Social economic factors, and subjective cognition. In terms of individual and family characteristics of farmers, the age, gender, educational background, breeding scale, and labor input of farmers will be the influencing factors of the response of livestock manure recycling behavior (Materechera, 2010; Gebrezgabher et al., 2015; Zhang and Xiao, 2016; Case et al., 2017). In terms of social and economic factors, government subsidies, social norms, whether to join cooperatives, environmental rules, and policies are all influencing factors in the response of livestock manure recycling behavior (Roubík et al., 2018; Li and Wang, 2019; Bulent gurbuz and Ozkan, 2021; Wang et al., 2021; Yao and Zhang, 2021; Yue et al., 2022). In terms of subjective cognition, value cognition, environmental knowledge, health belief, and so on will be the influencing factors of the behavioral response to the recycling of livestock manure (Afroz et al., 2009; Li and Wang, 2022; Yazdanpanah et al., 2022a; Zhang et al., 2022a).

The existing results have important reference significance and theoretical value for the research on the resource utilization behavior of livestock manure, but there are still some defects. Firstly, the behavior of livestock manure recycling is a decision-making process from intention initiation to behavior response. Secondly, the resource utilization of livestock manure has a high value, but whether the theoretical value can be truly transformed into the actual value will largely depend on the psychological perception of farmers. According to the theory of farmer behavior, perceived value is the most direct factor affecting individual willingness (Yazdanpanah et al., 2022a). The perceived value judgment of farmers will greatly affect their behavior of recycling livestock and poultry manure. Thirdly, with the development of economic diversification and rural modernization in China, the multiple occupations of farmers are more common. The concurrent operation of farmers will inevitably affect their choice of production mode and allocation of production factors (Zhong et al., 2016). The resource utilization of livestock manure is a process that requires long-term labor time and production factors. Therefore, different types of concurrent business may lead to different perceived values of livestock manure resource utilization behaviors by farmers, which will lead to different utilization behaviors. In the existing literature, farmers are assumed to be homogeneous groups with uniform behavior, ignoring the heterogeneity of the concurrent business.

To fill the above defects in the existing research and further enrich the relevant literature on the resource utilization of livestock manure, the innovation and marginal contribution of this paper are mainly reflected in the following aspects: Firstly, it takes the lead in introducing the theoretical basis of perceived value into the research, to explore how the perceived value drives farmers’ behavior of recycling livestock manure. Secondly, the decision-making process from intention initiation to behavioral response was connected in the study, and the decision-making mechanism of resource utilization of livestock manure was deeply explored. Thirdly, the multi-group structural equation is used in the study to explore the driving differences of perceived value theory in the process of livestock manure resource utilization among farmers with different types of concurrent businesses.

Finally, the research goal of this study is to provide scientific and effective policy and theoretical support for policymakers from the perspective of farmers’ psychological perception through field investigation and empirical analysis of the sample areas.

The structure of the remaining part of this paper is as follows: the second part analyzes the applied theory and puts forward the research hypothesis; The third part explains the selected data, variables, and models. The fourth part uses the model to analyze and discuss the data. The fifth part summarizes the research conclusions, puts forward policy suggestions, and expounds on the limitations of this research.

## 2. Theoretical analysis and research hypothesis

### 2.1. Theoretical analysis

**Figure 1:**
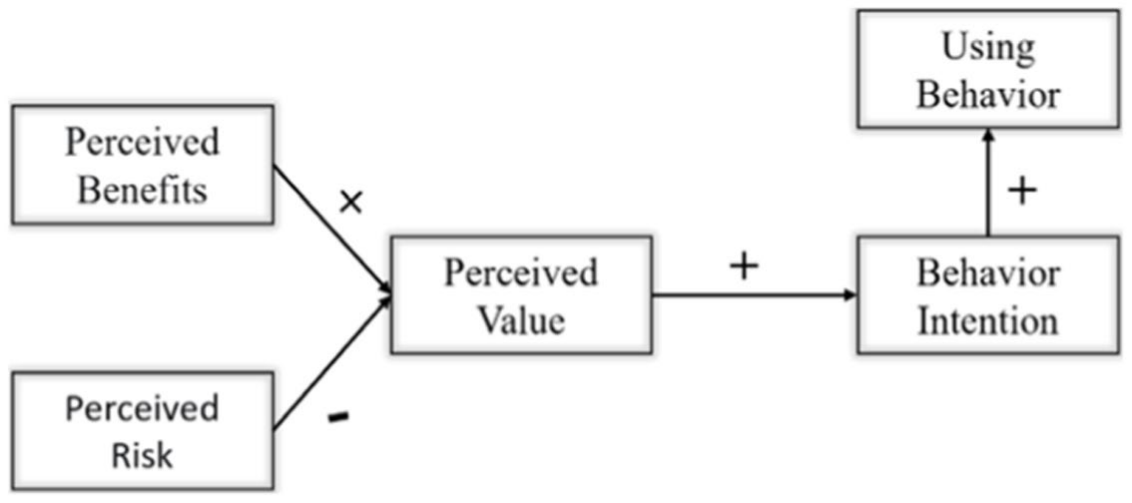
Theoretical analysis framework.

The theoretical model of perceived value was first proposed by Zeithaml in 1988 and should be used in the study of consumer behavior (Zeithaml, 1988). Perceived value refers to the overall evaluation of the behavior subject after comparing and weighing the perceived benefits and perceived risks brought by the behavior. After the behavioral subject perceives the perceived benefit as higher than the perceived risk, the overall perceived value level will be at a higher level, and then make the behavioral tendency more obvious. In essence, it is a refinement of the individual cognition of the actor (Sweeney and Soutar, 2001). In the existing research results, the theory of perceived value satisfies the path paradigm and logical mechanism of “cognitive level → cognitive trade-off → perceived value → behavioral intention → behavioral response” (Woodruff, 1997). This logical paradigm shows that individuals weigh the benefits and losses of their behaviors under the influence of self-cognition, to generate the self-perceived value of comprehensive evaluation to influence the individual’s behavioral willingness, and finally give rise to the individual’s behavioral response. Previous studies have proved that the perceived value theory has strong applicability in the research directions of farmers’ homestead withdrawal, farmers’ land input, green agricultural production, and farmers’ farmland protection (Ren et al., 2018; Hu et al., 2020; Li and Chen, 2020; Wang et al., 2022). The resource utilization behavior of livestock manure is a kind of agricultural subject behavior, which is essentially the result of the balance between the benefits and risks of the resource utilization of manure, and its path also satisfies the paradigm from cognition to behavior. In conclusion, this paper constructed a behavioral decision-making model for livestock manure resource utilization (Figure 1).

### 2.2. Research hypothesis

Perceived value includes perceived benefits and perceived risks. Perceived benefits refer to the amount of income that the subject perceives in the process of behavior, which has a positive impact on individual behavioral cognition (Tsujikawa et al., 2016). Perceived benefit has a positive effect on perceived value, that is, the higher the perceived benefit, the higher the perceived value. In this study, the benefits perceived by farmers can be divided into three dimensions: economic, social, and ecological. Farmers have higher perceived benefits when they perceive that the resource utilization of livestock manure can bring higher economic benefits or reduce part of production and breeding costs, reduce environmental pollution and disease transmission, and promote the construction of new countryside and the development of ecological civilization. Perceived risk refers to the subject’s negative perception of possible uncertain events in the process of behavior (Jacobs and Worthley, 1999), which has a significant negative impact on individual cognition (Leiserowitz, 2006). Perceived risk has a reverse effect on perceived value, that is, the higher the perceived risk, the lower the perceived value. In this study, the risks perceived by farmers can be divided into three dimensions: economy, technology, and policy. Farmers have higher perceived risks when they perceive that the resource utilization of livestock manure will involve more economic costs, require complex technical knowledge, and obtain low policy support and restrictions. The overall perceived value of farmers is the result of perceived benefits and perceived risks.

Behavioral intention refers to the psychological intention of the subject before the behavior. Theoretically, if the farmers hold a high perceived value for the resource utilization of livestock manure, it will stimulate their psychological intention to resource utilization of livestock manure and improve their behavioral intention. Utilization behavior refers to the input degree of the actor to the implementation of a behavior. Theoretically, the stronger the behavioral willingness of livestock manure resource utilization, the more likely it is for farmers to put their will into action, to improve the input intensity of farmers. To sum up, this paper makes the following assumptions:

*H1*: Perceived revenue has a positive driving effect on perceived value.

*H2*: Perceived risk has a reverse driving effect on perceived value.

*H3*: Perceived value has a positive driving effect on behavioral intention.

*H4*: Behavioral intention has a positive driving effect on using behavior.

## 3. Data, variables, and model

### 3.1. Data sources

The data in this study came from the field survey conducted by the research team in rural areas of Shandong Province from July to August 2022. The animal husbandry system in Shandong Province is complete and large scale. The annual production of poultry meat accounts for 1/6 of that in China, and the total production of meat, eggs and milk have been the first in China since 1992 (China Shandong, 2021)As a big breeding province in China, the annual output of livestock manure in Shandong Province also ranks among the top in China. The huge output of livestock manure not only becomes an important and difficult problem for rural human settlement environment improvement but also restricts the sustainable development of animal husbandry in Shandong Province. Therefore, the survey data based on Shandong Province are sufficiently representative and referential, which can meet the research needs of this paper.

**Figure 2:**
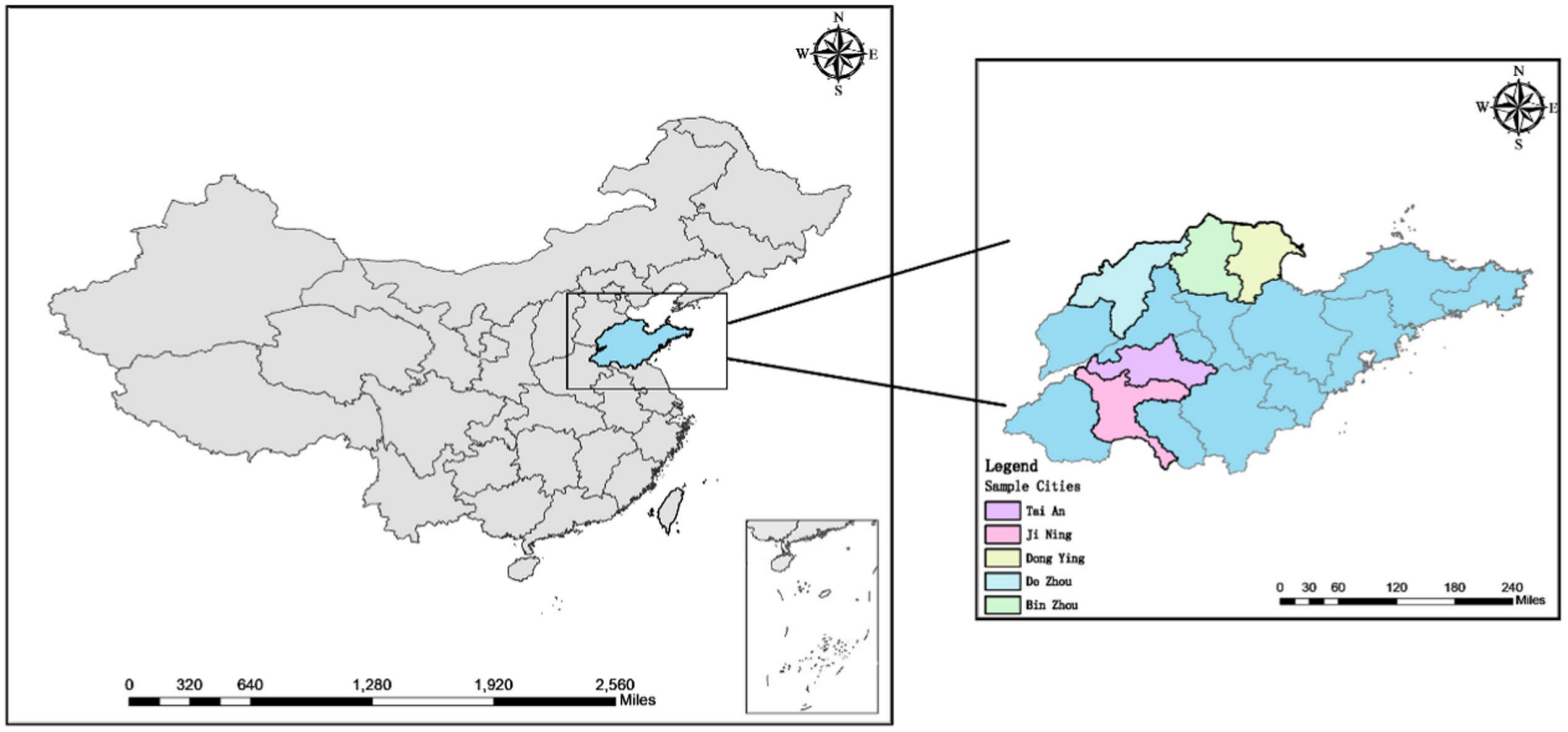
Survey region.

Our conduct research is divided into two categories, pre-survey and formal research, pre-survey is conducted by face-to-face interviews, and formal research is conducted by a combination of interviews and questionnaires. The pre-survey was conducted by face-to-face interviews with 20 randomly selected farmers in Jining City to get a preliminary understanding of the resource utilization of livestock and poultry manure by farmers, and the questionnaire was modified and improved according to the feedback results. In order to ensure that the sample was representative and could represent the overall population, the formal research used a combination of stratified sampling and random sampling. Firstly, five cities with large annual farming output in Shandong Province, Jining, Tai’an, Dezhou, Binzhou, and Dongying were selected, then two counties were randomly selected in each city, then 1–3 townships were randomly selected from each county, and finally the farmers to fill in the questionnaire were randomly selected and screened in these townships (Figure 2). Before conducting questionnaire research in each sampling area, the survey team first conducted face-to-face interviews with individual demonstration farmers under the leadership of local animal husbandry bureau staff, in order to understand the main local farming species and their manure treatment methods, etc., so that the subsequent questionnaire survey of other local farmers could be carried out smoothly. Pre-survey and interviews with demonstration farmers in each area took the same interview procedure steps. Interviews were conducted with the main person in charge of the farm and in most cases one or more family members or staff. The location of the interviews was chosen at the farm. The purpose of the interview was to ease the tension at the beginning of the interview, to reduce the pressure on the respondents to answer, and to make them feel comfortable so that the interview could be conducted properly and more detailed and realistic information could be obtained. The duration of the interviews ranged from 1 to 2 h. Through the interviews with the interviewees, we got a deeper understanding of the farmers’ personal and family situation and the real situation of perceived value. This provided a great help for the subsequent questionnaire research.

After interviewing the local model farmers, we conducted questionnaire research on other local farmers. Since face-to-face research can “reduce the response bias and improve the response rate” (Lou et al., 2021), we conducted face-to-face research at the farms or homes of the farmers. In the study, we first asked simple questions to the farmers, and those who answered the questions carefully were given a questionnaire to fill in. Before filling out the questionnaire, we declared to the farmers that their data would be kept confidential and they could choose voluntarily whether to fill out the questionnaire or not. Due to the low educational level of some farmers, we dictated the questions to those farmers who could not read or had difficulty in reading, without any bias guidance during the dictation process. The response time for each questionnaire was about 15 to 25 min. Finally, a total of 340 questionnaires were distributed and 340 were collected, of which 307 were valid and 33 were invalid, with an effective rate of 90.3%.

The distribution of individual characteristics of the sample is shown in Table 1. In terms of gender, men accounted for 80.8%, much higher than women who accounted for 19.2%, which may be due to the fact that livestock farming requires high-intensity physical labor, and men’s physical strength is higher than that of women. In terms of age group, 31.6% of the sample were under 44 years old, 52.8% were 44–59 years old, and 15.6% were over 60 years old, with a higher percentage of middle-aged and elderly people. There are two possible reasons for this, one is that young people go out to work, and during the survey, we found that the number of young people in the villages is significantly less than the number in the cities; the second is the result of increased aging, and China is currently in an aging stage. The education level of farmers is 76.5% in junior high school and below, and 23.5% in high school and above, which indicates that the education level of farmers is not high. The proportion of labor input 1–3 people is 70.36%, the proportion of input 4–7 people is 27.69%, and the proportion of input more than 7 people is 1.95%, which indicates that most of the farmers are mainly family farming.

**Table 1:** Main characteristics of the sample.

Type	Option	Quantity	Proportion	Type	Option	Quantity	Proportion
Sex	Male	248	80.8	Education level	Primary school and below	235	76.5
	Female	59	19.2		Junior high school and above	72	23.5
Age	Under 44	97	31.6	Labor input	1–3	216	70.4
	45–59	162	52.8		4–7	85	27.7
	Over 59	48	15.6		Over 7	6	1.9

### 3.2. Variable definition

#### 3.2.1. Behavior of resource utilization of livestock manure

Referring to the research of Ren and Zhong (2022) and Boz (2016), the utilization decision and utilization intensity were used to characterize the resource utilization behavior of livestock manure of farmers. Since the most direct form of resource utilization behavior of farmers is “yes” and “no,” the utilization decision adopts the binary assignment method. If the farmer has carried out the resource utilization of livestock manure, the value is 1, if not, the value is 0. Utilization intensity refers to the number of years for livestock manure resource utilization.

#### 3.2.2. Behavioral intention of livestock manure recycling

Referring to the research of Li Q. et al. (2021), Li (2012), and other scholars, the behavioral intention of livestock manure recycling was characterized by three aspects: Recommendation intention, utilization intention, and investment intention. All items were measured on a 5-point Likert scale. The answer options were “very low,” “low,” “general,” “high,” and “very high,” with the values of “1–5,” respectively.

#### 3.2.3. Perceived value of livestock manure resources

Referring to the research of Li et al. (2020), Ren et al. (2018), and other scholars, the perceived value of livestock manure resources was characterized by three aspects: Value cognition, behavior attitude, and recommendation cognition. All items were measured on a 5-point Likert scale. The answer options were “very low,” “low,” “general,” “high,” and “very high,” with the values of “1–5,” respectively.

#### 3.2.4. Perceived risk of livestock manure resource utilization

Referring to the research of Wang et al. (2022) and Jin et al. (2022), the perceived risks of livestock manure recycling were characterized by three aspects: economic risk, technical risk, and policy risk. All the items were measured on a 5-point Likert scale, and the answer options were “completely disagree,” “disagree,” “basically agree,” “relatively agree,” and “completely agree,” with the values of “1–5,” respectively.

#### 3.2.5. Perceived benefits of livestock manure resource utilization

Referring to the research of Wang et al. (2019) and Li et al. (2020), the perceived value of livestock manure resource utilization was characterized by three aspects: economic benefits, ecological benefits, and social benefits. All the items were measured on a 5-point Likert scale, and the answer options were “completely disagree,” “disagree,” “basically agree,” “relatively agree,” and “completely agree,” with the values of “1–5,” respectively (Table 2).

**Table 2:** Variable definition and measurement items.

Variable	Index	Measurement items	Standard deviation
Using behavior (UB)	Using decision	Whether the fecal waste resources have been used (UB_1_)	0.367
	Using intensity	The number of years of continuous fecal recycling (UB_2_)	1.277
Behavior intention (BI)	Recommendation intention	Willing to recommend to others the degree of resource utilization of livestock manure (BI_1_)	0.935
	Utilization intention	The degree of willingness to recycle livestock manure (BI_2_)	0.981
	Investment intention	Willing to invest a certain amount of money, time, labor and other costs in the process of recycling livestock manure (BI_3_)	1.075
Perceived value (PV)	Behavior attitude	Positive attitude toward resource utilization of livestock manure (PV_1_)	0.940
	Value cognition	It is believed that the resource utilization of livestock manure can bring certain benefits (PV_2_)	0.912
	Significance cognition	It is considered that the resource utilization of livestock manure has positive significance (PV_3_)	0.922
Perceived risk (PR)	Economic risk	Concerned about the excessive labor, time, and money involved in the processing process (PR_1_)	0.815
	Technical risk	Concerned about not being able to master the required knowledge and techniques (PR_2_)	0.823
	Policy risk	Concerned that the government’s relevant policy formulation and implementation is not in place (PR_3_)	0.852
Perceived benefits (PB)	Economic benefits	Can reduce breeding cost, raise income level (PB_1_)	0.998
	Ecological benefits	Can reduce the spread of disease and protect the ecological environment (PB_2_)	0.958
	Social benefits	Can promote ecological progress and the development of a new countryside (PB_3_)	0.954

### 3.3. Model construction

In the setting of this study, variables such as perceived benefits, perceived risks, and perceived values are all latent variables and their degrees are difficult to directly observe. Structural Equation Model (SEM), also known as latent variable model, is a statistical method using linear equation system to express the relationship between observed variables and latent variables, as well as between latent variables and is widely used in psychology and social sciences. It has the advantage of not only its strong adaptability to the measurement of latent variables, but also its ability to deal with multiple dependent variables at the same time, estimate factor structure and factor relationship, and better reveal the relationship between variables. Structural equation model can be combined with theoretical model to meet the requirements of scholars, such as Theory of Planned Behavior (Yazdanpanah et al., 2022b), Theory of Reasoned Action (Senger et al., 2017), Theory of Perceived Value (Li et al., 2020), etc. In addition, there are many literatures using SEM to conduct researches on farmers. Therefore, this paper combines structural equation model with perceived value theory to study. The equation is expressed as follows:


(1) η=Βη+Γξ+ζ

(2) Y=Λyη+ε

(3) X=Λxξ+δ

Equation (1) is the structural equation, η is the endogenous latent variable; Β is the coefficient of endogenous latent variable η. ξ is an exogenous latent variable; Γ is the coefficient of the exogenous latent variable ξ. ζ is the residual. Equations (2) and (3) are measurement equations, Y and X are observed variable vectors of endogenous latent variable η and exogenous latent variable ξ, respectively. Λy and Λx represent the correlation coefficient matrix of Y on η and X on ξ, respectively. Both ε and δ represent measurement errors.

Considering the heterogeneity of concurrent business among farmers, this paper uses the type of concurrent business of farmers as the moderating variable to conduct a multi-group analysis, which can better test the driving effects of perceived value on the resource utilization behavior of livestock and poultry waste of different concurrent business types of farmers. Multi-group SEM analysis is to evaluate whether the model adapted to a certain text is also adapted to other different sample groups, that is, to evaluate whether the hypothetical model proposed by the researcher is equal between different samples or whether the parameters have invariability (Cui and Li, 2018). Based on Li and Zhao’s (2017) classification standard for the type of concurrent business, this paper made adjustments according to the actual investigation situation, and divided the farmers into two types: full-time farming and combined farming, so as to facilitate the multi-group SEM analysis. Among them, full-time farming refers to the farmers whose main income of livestock and poultry farming accounts for 80% or more of the total income, and the combination type refers to the farmers whose family livestock and poultry farming account for less than 80% of the family income. In the survey, the proportion of combined cultivation and full-time cultivation was 71.7% and 28.3%, respectively.

## 4. Data analysis and discussion

### 4.1. Reliability and validity test

To ensure the validity and credibility of the research data, SPSS26.0 was used to test the overall reliability and validity of the questionnaire and the reliability and validity of each latent variable, respectively. The test results showed that the Cronbach’s α value of the overall reliability of the questionnaire was 0.786, and the Cronbach’s α values of PB, PR, PV, BI, and BR were 0.720, 0.635, 0.788, 0.756, and 0.608, respectively, which were all higher than the standard value 0.6. The KMO value of the overall validity of the questionnaire was 0.837, which was higher than the benchmark value of 0.7, and the KMO value of each latent variable was also higher than the benchmark value of 0.5, indicating that the data had good reliability and validity. In addition, Harman univariate test technique was used to conduct principal component analysis to test the common method bias. Principal component analysis was carried out for each item. It was found that the characteristic root of three factors was greater than 1, and the variance explanation rate of the first common factor was 35.7% < 40%. According to the indexes of the Harman univariate test results, there is no serious common method bias effect among the variables. The results were within the acceptable range (Shiau and Luo, 2012). The specific convergent validity information of the experimental data is shown in Table 3.

**Table 3:** Convergent validity table.

Variables	Items	Significance estimation			Reliability of questions		Component reliability	Convergent validity
		S.E.	*z*-value	*P*	Std.	SMC	CR	AVE
Perceived benefits	PB_1_				0.616	0.379	0.727	0.472
	PB_2_	0.131	8.831	***	0.739	0.546		
	PB_3_	0.126	8.658	***	0.7	0.49		
Perceived risk	PR_1_				0.661	0.437	0.636	0.369
	PR_2_	0.151	6.075	***	0.599	0.359		
	PR_3_	0.147	6.012	***	0.558	0.311		
Perceived value	PV_1_				0.709	0.503	0.78	0.543
	PV_2_	0.09	10.507	***	0.69	0.476		
	PV_3_	0.095	11.716	***	0.807	0.651		
Behavior intention	BI_1_				0.635	0.403	0.734	0.481
	BI_2_	0.126	9.876	***	0.756	0.572		
	BI_3_	0.133	9.295	***	0.684	0.468		
Using behavior	BR_1_				0.874	0.764	0.895	0.81
	BR_2_	0.223	16.548	***	0.925	0.856		

*** *p* < 0.01.

### 4.2. Model fitness test

In order to judge the degree of fit between the research reality and the null hypothesis, AMOS23.0 software was used to test the fitness of the theoretical model. The test results show that the model has good significance and meets the adaptation standard, and has a good model fitness. The detailed data are shown in Table 4.

**Table 4:** Fitting results of model fitness.

Goodness-of-fit index	Absolute fit index				Value-added compatibility indicators			Simple fit index		
	χ2/df	GFI	AGFI	RMSEA	TLI	CFI	IFI	PNFI	PCFI	PGFI
Criteria	<3	>0.9	>0.8	<0.08	>0.8	>0.9	>0.9	>0.5	>0.5	>0.5
Modified fitting effect	2.897	0.917	0.879	0.079	0.889	0.912	0.913	0.691	0.722	0.629

### 4.3. Structural equation model estimation result

**Figure 3:**
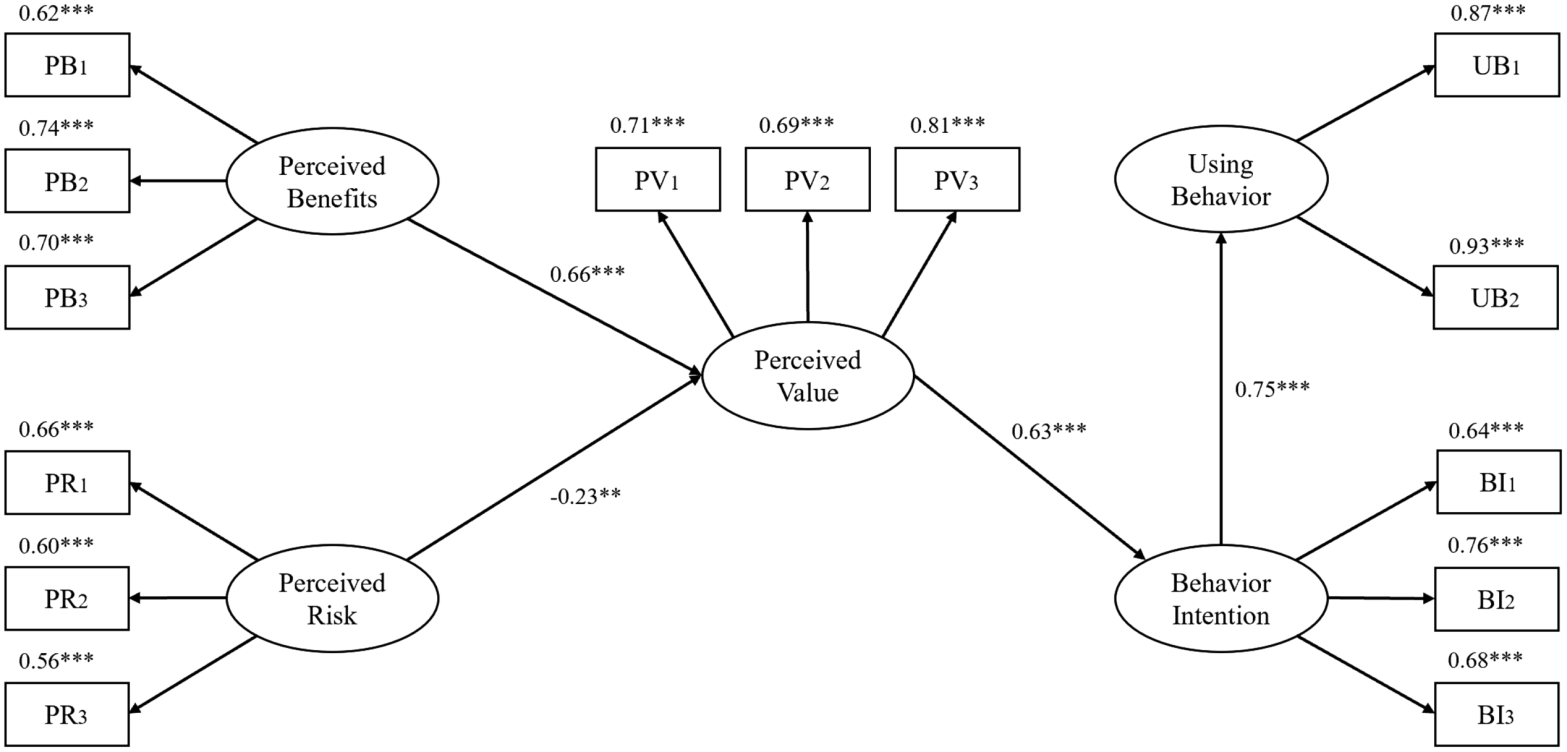
Structural equation model and standardized path coefficient diagram. ^**^p < 0.05; ^***^p < 0.01; PB, Perceived Benefits; PR, Perceived Risk; PV, Perceived Value; UB, Using Behavior; BI, Behavior Intention.

After the model calculation, it was found that the livestock manure resource utilization behavior of farmers followed the logic of “cognitive level → cognitive trade-off → perceived value → behavioral intention → behavioral performance.” All hypotheses H1–H4 above hold. As shown in Figure 3 (*p < 0.10, ** *p* < 0.05, *** *p* < 0.01, PB, Perceived benefits; PR, Perceived risk; PV, Perceived value; BI, Behavioral intention; UB, Using behavior).

#### 4.3.1. Perceived benefits

Perceived benefit has a positive driving effect on perceived value, and its path coefficient is 0.66, which is larger than that of perceived risk, indicating that its driving effect on perceived value is more obvious. Among the three observed variables of perceived benefits, the path coefficients of economic benefits, ecological benefits, and social benefits are 0.62, 0.74, and 0.70, respectively, which indicates that ecological benefits have the greatest impact on the perceived benefits of farmers. This is consistent with the conclusion proved by Bin et al. that “farmers show strong positive awareness of the ecological benefits of positive externalities” (Bin et al., 2017). According to the principle of rational economic man hypothesis, economic benefit should be the factor that has the greatest impact on perceived benefit. But in the survey found that due to the livestock manure resource utilization system is not perfect, their use or resources into the sale of income is not high, most farmers can only be reached after livestock manure recycling use or a slight surplus of balance of payments, but after waste resource recovery of ecological environment, quality and public health improvement is more apparent.

#### 4.3.2. Perceived risk

Perceived risk has a reverse driving effect on perceived value, and its path coefficient is −0.23. Among the three observed variables of perceived risk, the path coefficients of economic risk, technical risk, and policy risk are 0.66, 0.60, and 0.56, respectively, and the path coefficient of economic risk is the largest, which indicates that economic risk has the greatest influence on farmers’ perceived risk. This is consistent with the conclusion confirmed by Lin et al. that “economic affordability is the most important factor for farmers to consider when polluting livestock manure (Lin et al., 2018). It is found that due to the frequent occurrence of animal diseases in recent years, the risk of livestock and poultry breeding is large, and the cost of all kinds of breeding is high. In addition, the process of resource utilization of livestock manure requires a lot of labor, time, capital and other inputs. Farmers will be concerned about the various costs generated by the resource utilization of livestock manure. And affected by the total number of farmers with the combination of breeding and breeding, a large number of farmers not only have to carry out livestock breeding but also have to carry out agricultural production, so the impact of farmers on labor and time investment is also at a low level.

#### 4.3.3. Perceived value

Perceived value has a positive driving effect on behavioral intention, and its path coefficient is 0.63. Among the three observed variables of perceived value, the path coefficients of behavior attitude, value cognition, and meaning cognition are 0.71, 0.69, and 0.81, respectively, and the path coefficient of perceived meaning cognition is the largest, which indicates that meaning cognition has the greatest influence on perceived value. This is consistent with the conclusion confirmed by Li et al. (2020). Found in the field survey, policy propaganda focuses on livestock manure recycling behavior in environmental protection, public health, and the importance of social construction. In addition, the supervision and regulation of village cadres in the implementation process gives farmers a sense of urgency, which leads to a higher cognition of the significance of the livestock manure resource utilization, but a lower cognition of value.

#### 4.3.4. Behavioral intention

Behavioral intention had a positive effect on utilization behavior, and the path coefficient was 0.75. The stronger the willingness of farmers to utilize livestock manure pollution resources, the more likely the utilization behavior was. Among the three observed variables of behavioral intention, the path coefficients of promotion intention, utilization intention, and investment intention were 0.64, 0.76, and 0.68, respectively, and the path coefficient of utilization intention was the highest, which indicated that the overall farmers still had a relatively positive attitude toward the resource utilization of livestock manure, and were willing to try and participate in it. But relatively speaking, farmers are not willing to invest too much economic, time, and labor costs.

### 4.4. Multi-group model test

In multi-group analysis, various parameter restrictions are needed to find out the most suitable path model (Lee and Whittaker, 2021). After comparing the adaptation indexes of the baseline model (i.e., the unconstrained model), the measurement weights model, the structural weights model, the structural covariances model, the structural residuals model, and the measurement residuals model. The measurement weights model is finally selected as the multi-group analysis model in this paper, and the results are shown in Table 5.

**Table 5:** Multi-group analysis adaptation results.

Model	CMIN	DF	*P*	CMIN/DF	CFI	RMSEA	AIC	ECVI	PCFI
Unconstrained	267.135	144	0	1.855	0.906	0.053	399.135	1.309	0.717
Measurement weights	284.484	153	0	1.859	0.899	0.053	398.484	1.307	0.756
Structural weights	313.719	157	0	1.998	0.88	0.057	419.719	1.376	0.759
Structural covariances	333.081	160	0	2.082	0.868	0.06	433.081	1.42	0.763
Structural residuals	376.59	163	0	2.31	0.837	0.066	470.59	1.543	0.749
Measurement residuals	407.812	177	0	2.304	0.823	0.065	473.812	1.553	0.801

The invariance test was carried out after the indexes of each model were well-matched. Comparing the five models with the baseline model, the results in Table 6 show that ΔP is less than 0.05, indicating that there are significant differences in the corresponding sample models of different project types (Zhang et al., 2022b).

**Table 6:** Results of the invariance test.

Model	Delta-CMIN	Delta-DF	Delta-P	Delta-CMIN/DF	Delta-CFI	Delta-RMSEA	Delta-AIC	Delta-ECVI	Delta-PCFI
Measurement weights	17.349	9	0.044	0.004	−0.007	0	−0.651	−0.002	0.039
Structural weights	46.584	13	0.000	0.143	−0.026	0.004	20.584	0.067	0.042
Structural covariances	65.946	16	0.000	0.227	−0.038	0.007	33.946	0.111	0.046
Structural residuals	109.455	19	0.000	0.455	−0.069	0.013	71.455	0.234	0.032
Measurement residuals	140.677	33	0.000	0.449	−0.083	0.012	74.677	0.244	0.084

As shown in Table 7, many groups of samples are similar to the results of the analysis of the whole samples: the perceived benefits and perceived risk to the perceived value and perceived value to the behavior intention and behavior intention of using action behavior have a significant role in driving in the path, and the direction the same as the whole sample analysis, hypothesis H1–H4 in different types of farmers has been proved again. However, there are some differences, mainly showing that the driving strength of perceived benefit and perceived risk to perceived value is significantly different for different farmer types. In the full-time farming group, the driving effect of perceived benefit on perceived value, the driving effect of perceived risk on perceived value, the driving effect of perceived value on behavioral intention, and the driving effect of behavioral intention on utilization behavior were stronger than those in the group of combination planting and breeding. This is consistent with the previous conclusion obtained by Zhang et al. (2022b). The possible reason is that full-time farmers have a higher degree of farming specialization and are less dependent on land production. The survey found that in the process of livestock manure recycling use full-time farmers’ investment of capital, technology and equipment is more, by using the process toward large scale, and most full-time farmers farming scale is larger, the government subsidy and support degree is bigger, the results of that waste recycling use have a good sale and use of a way out. On the contrary, the farmers of the combination type have a weak perception of technology access, policy subsidies, and significance value, which may lead to a low behavioral intention of the combination type farmers.

**Table 7:** The result of Multi-group model test.

Affect the path	Full-time farming	Combination cultivation and breeding
Perceived value ← perceived benefits	0.817^***^	0.404^***^
Perceived value ← perceived risk	−0.380^**^	−0.226^**^
Behavioral intention ← perceived value	0.716^***^	0.366^***^
Using behavior ← behavioral intention	0.713^***^	0.632^***^

** *p* < 0.05.

*** *p* < 0.01.

## 5. Conclusion

### 5.1. Conclusion

Based on the survey data of farmers in Shandong Province of China in 2022, the multi-group structural equation model was used to empirically analyze the driving mechanism of perceived value on livestock manure resource utilization behavior of farmers. The following conclusions are obtained:

1. Livestock manure resource utilization behavior of farmers follows the logic of “cognitive level → cognitive trade-off → perceived value → behavioral intention → behavioral performance.” It shows that perceived benefit and perceived risk have positive and reverse driving effects on perceived value, respectively. Perceived value has an obvious positive driving effect on behavioral intention. The behavioral intention has an obvious positive driving effect on utilization behavior.
2. Among the observed variables of perceived benefits, ecological benefits have the greatest impact; Among the observed variables of perceived risk, economic risk has the greatest influence. Among the observed variables of perceived value, meaning cognition has the greatest influence. Among the observed variables of behavioral intention, intention to utilize has the greatest influence.
3. Perceived value had differentiated effects on livestock manure resource utilization behavior of different part-time farmers, and its driving effect on full-time farmers was more obvious.

### 5.2. Policy recommendations

Based on the above research conclusions, the following policy recommendations are put forward:

1. Since perceived income and perceived risk have significant positive and negative driving effects on perceived value, respectively, and economic income has the lowest impact on perceived income, while economic risk has the largest impact on perceived risk, it is necessary to improve the resource utilization system of livestock manure so that the products of livestock and poultry manure resource utilization can have a better realization channel, to solve the situation that the income and expenditure of livestock and poultry manure resource utilization by farmers offset or have a slight surplus; We should strengthen the investment in science and technology, reduce the operating cost of technical facilities, and achieve the goal of reducing the high investment in the economy, time, and labor in the use of farmers; Formulate incentive and subsidy policies, improve industry operation laws and regulations, to improve the perceived value of farmers in general.
2. Since perceived value has a significant positive driving effect on behavioral will, behavioral will has a significant positive driving effect on utilization behavior, value cognition has the least impact on perceived value, and recommendation intention has the least impact on behavioral will, we should make full use of various information dissemination drivers to increase the significance and value publicity of livestock and poultry manure recycling. In the process of publicity, attention should be paid to the multi-dimensional development, not only to enable farmers to realize the significance and value of resource utilization of livestock and poultry manure to society but also to make them feel the significance and value of resource utilization to themselves, to improve the sense of identity and acquisition of farmers.
3. Since perceived value has a stronger driving effect on full-time farmers and a weaker driving effect on the combination of planting and farming farmers, the promotion of livestock manure resource utilization needs to be “classified and implemented.” For the combination type farmers, policy subsidies should be increased and the access threshold of technology and equipment should be reduced. Full-time farmers, can be encouraged to actively try new treatment methods to promote more convenient and effective livestock manure resource production.

### 5.3. Research limitations and prospects

Finally, it should be pointed out that this study still has some limitations. (1) Survey region: in this paper, the data of farmers in Shandong, China, are used, so the sample coverage is small and the survey area is narrow. Due to the in-depth economic diversification and regional development differences and other factors, it is necessary to try to further verify and analyze the data of farmers at the national level or in areas with obvious economic development degrees. (2) The division of the types of farmers’ part-time jobs: in this paper, the types of part-time farmers are directly divided into two types: the combination of planting and farming and full-time farmers, and the degree of fineness of division still needs to be improved. Future studies can classify the types of farmers according to more detailed standards, to study the differences in the resource utilization behavior of livestock manure pollution of different types of farmers. (3) The data in this study are from cross-sectional data, which cannot investigate the long-term dynamic change process of farmers’ resource utilization behavior. In the future, time series data should be established to conduct a more comprehensive and systematic analysis of farmers’ resource utilization behavior.

## Data availability statement

The raw data supporting the conclusions of this article will be made available by the authors, without undue reservation.

## Ethics statement

The studies involving human participants were reviewed and approved by Shandong Normal University. The patients/participants provided their written informed consent to participate in this study.

## Author contributions

RG and GL built the conceptual model of the paper and analyzed the data. XW and YF conducted field investigations and collected data. RG wrote the paper. ZR put forward the idea of writing the paper and carried on the guidance and revision to the paper. All authors contributed to the article and approved the submitted version.

## Conflict of interest

The authors declare that the research was conducted in the absence of any commercial or financial relationships that could be construed as a potential conflict of interest.

## Publisher’s note

All claims expressed in this article are solely those of the authors and do not necessarily represent those of their affiliated organizations, or those of the publisher, the editors and the reviewers. Any product that may be evaluated in this article, or claim that may be made by its manufacturer, is not guaranteed or endorsed by the publisher.
